# Advancing plant disease classification using an attention-based CNN for intra-dataset and cross- dataset training

**DOI:** 10.1038/s41598-026-45464-7

**Published:** 2026-03-27

**Authors:** Prateek Mahapatra, Madhumita Panda, Santanu Kumar Dash, Umesh Kumar Sahu

**Affiliations:** 1 School of Computer Science, Gangadhar Meher University , Sambalpur, Odisha India; 2 TIFAC-CORE, Vellore Institute of Technology, Vellore, Tamil Nadu 632014 India; 3 Manipal Institute of Technology, Manipal Academy of Higher Education, Manipal, India

**Keywords:** Plant disease, Cross, Dataset, Intra, Dataset, Classification, Deep learning, Attention, Based CNN, Feature extraction, Precision farming

## Abstract

The precise classification of plant diseases is crucial for ensuring food security for all people and boosting agricultural productivity. Although there has been significant progress in this field using deep learning approaches, cross-dataset training hasn’t drawn as much attention from researchers as intra-dataset training has. Moreover, very few models have successfully blended intra-dataset and cross-dataset training approaches. This paper proposes a novel attention-based Convolutional Neural Network (CNN) to overcome these limitations. The model improves feature extraction and classification accuracy across multiple datasets by using attention mechanisms. It was tested on five datasets (Digipathos, Northern Leaf Blight (NLB), PlantVillage, PlantDoc, and the CD&S dataset) that covered leaf diseases of both corn and potatoes. During intra-dataset training, the model achieved the highest classification accuracy of 99.38% when trained on images of potato leaves from the PlantVillage dataset. During cross-dataset training, the model exhibited the highest average classification accuracy of 82.93% for corn leaf diseases when trained on images from the CD&S dataset with their backgrounds removed. When compared to the techniques taken into consideration in this study under comparable experimental conditions, the results demonstrate improved performance. This study shows how the model may be flexible for both intra- and cross-datasets, offering a flexible way to categorize diseases that affect plants. Because of its ability to generalize across different datasets, it may be helpful in real-world agricultural applications with a wide variety of image quality and situations. This encourages the advancement of precision farming techniques and disease control.

## Introduction

The accurate and timely classification of plant diseases is crucial to ensuring global food security and agricultural output. Climate change, disease outbreaks, and rising global food demand are all putting additional strain on agricultural systems, making effective plant disease management solutions more important than ever. Traditional disease detection methods, which rely on expert human observation, are not only time-consuming but also error-prone, particularly when disease symptoms are mild or similar across plant species^1^. Advanced hybrid approaches have been designed to address the challenges and limitations of traditional manual plant disease detection^2^. Improvements in machine learning, particularly deep learning, have shown great promise in automating and improving the accuracy of plant disease classification.

Convolutional Neural Networks (CNNs) have emerged as a powerful tool for image-based plant disease detection, with outstanding results on single datasets^3^. Various deep learning techniques, such as the Gradient Weighted DenseNet-201 (GradWDN-201) CNN, combine DenseNet-201 with Gradient Weighted Class Activation Mapping, and image texture features are also used^4^. Models like the ExCNN-LFCP, which combine Extra Convolutional Neural Networks with Levy Flight Carnivorous Plant optimization and IoT integration, have also been employed to enhance environmental monitoring and agricultural efficiency^5^. These algorithms can identify hidden patterns in leaf symptoms, resulting in high classification accuracy. Despite significant advances in the development of deep learning-based models for plant disease classification, the majority of research has concentrated on intra-dataset training, which includes training and testing the model on images from the same dataset. This strategy limits the model’s generalization ability, as models trained solely on intra-dataset data usually perform poorly when exposed to different datasets^6^. Cross-dataset training and evaluation, which assesses a model’s generalization ability across many datasets, is a relatively unexplored topic in plant disease detection^7^.

Attention mechanisms offer a possible answer to these limitations, allowing models to focus on the most significant parts of an image. These techniques have made significant progress in image classification tasks in sectors such as medical imaging and agricultural analysis^8^. In the instance of plant disease identification, including attention processes in CNNs may improve the model’s ability to extract key properties, resulting in increased classification accuracy in both intra- and cross-datasets. This study proposes a novel attention-based CNN model that enhances feature extraction and classification performance across a variety of datasets. By including attention processes, the model can focus on the most relevant visual regions, increasing classification accuracy. The model was tested on five distinct datasets (PlantVillage, PlantDoc, Digipathos, Northern Leaf Blight (NLB), and CD&S), which included corn and potato leaf images. Preliminary results show that the model is very adaptive and accurate, with potential for intra- and cross-dataset evaluations. This adaptability is crucial for increasing the robustness of plant disease classification algorithms in real-world agricultural applications, where climatic conditions and image quality can vary substantially. This study is organized into six parts. Part 2 offers a brief overview of related studies. Part 3 outlines the methodology. Part 4 presents and analyzes the results. Limitations of the study are reported in part 5. The final part, part 6, concludes the paper and suggests potential future directions for research.

## Related work

The development of automated systems for image-based classification of plant leaf diseases has recently received a lot of interest. Researchers used a variety of deep learning techniques, including image preprocessing, convolutional neural networks (CNNs) for feature extraction, and advanced plant leaf disease classification models. The goal of this methodology is to improve the accuracy and effectiveness of plant disease detection and classification.

Some of the researchers had also used an attention mechanism with CNN. A study^9^ proposed a Wild Horse Optimizer–based Convolutional Attention Bidirectional Long Short-Term Memory (BiLSTM) model for plant leaf disease classification. By integrating CNN, attention, and BiLSTM layers, it effectively identified diseased leaves using the PlantVillage dataset with 97.55% accuracy. The model reduces overfitting and enhances generalization.

An Attention Convolutional Bidirectional Gated Recurrent Unit (BiGRU) model combined with a Modified Leaf-in-Wind optimizer integrates CNN feature extraction, temporal BiGRU context, and attention weighting for disease detection and environmental monitoring across PlantVillage, PlantDoc, soil and IoT datasets, achieving 97.5–98.5% in four scenarios; the approach boosts accuracy^10^. So, from this, we know that attention mechanisms are quite helpful with CNN for classifying plant leaf diseases.

In this study^6^, leaf images of the PlantVillage dataset were used for training and then evaluated in an actual field setting achieving a testing accuracy of 33.27% only. On the other hand, testing accuracy increased to 65.69% when field- based environment leaf images were used for training and testing on the PlantVillage dataset, demonstrating difficulties with cross-dataset generalization.

The study^11^ demonstrated an enhanced Faster-RCNN technique for deep keypoint computation that incorporated ResNet-50 and spatial-channel attention. The MaizeNet model performed admirably in recognizing and categorizing corn leaf diseases, with an average accuracy of 97.89% and a mean relative precision of 0.94 on the Corn Disease and Severity dataset.

In this study^12^, transfer learning was applied using five previously trained deep neural networks: InceptionV3, ResNet50, VGG16, DenseNet169, and Xception with removed background images from the CD&S dataset. DenseNet169 performed best for corn disease classification, with an accuracy of 87.52%. Using the PlantVillage, PlantDoc, Northern Leaf Blight (NLB), Digipathos, and CD&S datasets, this study also performed both intra- and cross-dataset training for the categorization of corn diseases.

This study^13^ investigated the identification of corn leaf diseases using deep learning models and the PlantVillage dataset. By incorporating attention techniques into MobileNetV2 and VGG16, accuracy was improved. The 94.76% accuracy of MobileNetV2 and the 93.44% accuracy of the VGG16 model using Squeeze-and-Excitation (SE) blocks allowed for better agricultural outcomes and early disease diagnosis.

The entire PlantVillage dataset, which also contains images of corn leaves, was used in this study^14^ to detect plant diseases using deep learning and feature extraction techniques. The approach’s efficacy in precisely recognizing diseases in various plant species was demonstrated by utilizing convolutional neural networks (CNNs), which achieved an accuracy of 97.82%.

To identify plant diseases using images from the PlantVillage dataset, this study^15^ used a conditional multi- task learning strategy utilizing a ResNet-based model. Its average accuracy of classification was 94.52%.

In this study^16^, the VGG16 model, which was trained on the PlantVillage dataset, was tested on the PlantDoc dataset attaining an accuracy rate of 39.87%. This study also established the PlantDoc dataset as a visual aid for plant disease identification. The dataset contains diseased leaf images of plants such as tomatoes, potatoes, corn, and peppers. Their study classified diseased leaves using convolutional neural networks (CNNs) with an accuracy of about 57%. The development and evaluation of effective illness detection models was facilitated by this dataset.

In this study^17^, they introduced the EfficientPNet model, designed for accurate and computationally efficient classification of potato leaf diseases. The algorithm was trained and evaluated on the PlantVillage dataset, which includes annotated pictures of potato leaves with multiple diseases. EfficientPNet obtained 98.12% accuracy, demonstrating its usefulness in precision agriculture for early disease detection and management in potato crops.

By fine-tuning the Xception model, the study^18^ achieved 95.37% accuracy in potato leaf disease classification. Using the PlantVillage dataset, it revealed that advanced CNN architectures improve disease detection performance.

The PlantDoc dataset^19^ was not extensively utilized in the scientific community due to its small size. It was mostly used for duties involving the identification of illnesses.

A few new datasets, including the Digipathos^20^ and NLB^21^, were made available to the general public. The NLB dataset exclusively featured photos of the Northern Leaf Blight disease on corn. The Digipathos dataset includes leaf images of various plants, also comprising diseased leaf images of corn.

MobileNet, VGG16, InceptionResNetV2, and InceptionV3 models were used in this study^22^ to train across datasets. When trained on the PlantVillage dataset and tested on the PlantDoc dataset, InceptionResNetV2 had the highest average classification accuracy of 17.32%. Leaf images of corn and tomato were used in this study.

In this study^23^, the model trained on lab-based images achieved 85% accuracy when evaluated on similar lab images, but only 61% when tested on-field images. In contrast, the model trained on field-based images obtained 73% accuracy, but just 58% when tested on lab images. This highlighted the difficulties of cross-dataset generalization between lab and field contexts for crop disease detection.

In this study^24^, the VGG16 model was employed for cross-dataset training. When this model was trained on the PlantVillage dataset and evaluated on the IPM dataset, it scored 45.95%. However, when the same model was evaluated on the Bing dataset, its accuracy fell to 33.97%. This highlighted the limitations of applying a model trained in controlled settings to real-world photos of plant diseases.

In this study^25^, the ResNet-50 model, which was trained on the PlantVillage dataset, was tested on two sets of field-based environmental datasets. On these datasets, the model attained accuracy rates of 39.38% and 72.03%, respectively.

The study^26^ introduced the PlantVillage dataset, which has been famous for quite some time, in the diagnosis of diseases of both corn and potato crops.

In this study^27^, a customized convolutional neural network (CNN) model was developed to identify corn disease. Preprocessing techniques such as log transformation, RGB to HSV conversion, and Contrast Limiting Adaptive Histogram Equalization (CLAHE) on RGB channels were included. These models’ efficacy was compared to that of Support Vector Machine (SVM) and non-preprocessed CNN models. The study illustrated the impact of preprocessing on model performance with a 96.76% classification accuracy using images of corn from the PlantVillage dataset.

To summarize, whereas many studies have focused on plant disease classification using intra-dataset training, research on cross-dataset training is limited. The majority of models are tuned for specific datasets and cannot successfully generalize across a wide range of datasets. Furthermore, few studies have successfully merged intra- and cross-dataset training in a single model. This study seeks to bridge this gap by proposing an attention-based CNN model that enhances intra- and cross-dataset performance, resulting in a more robust solution for plant disease classification.

## Proposed methodology

### Datasets utilized

**Fig. 1:**
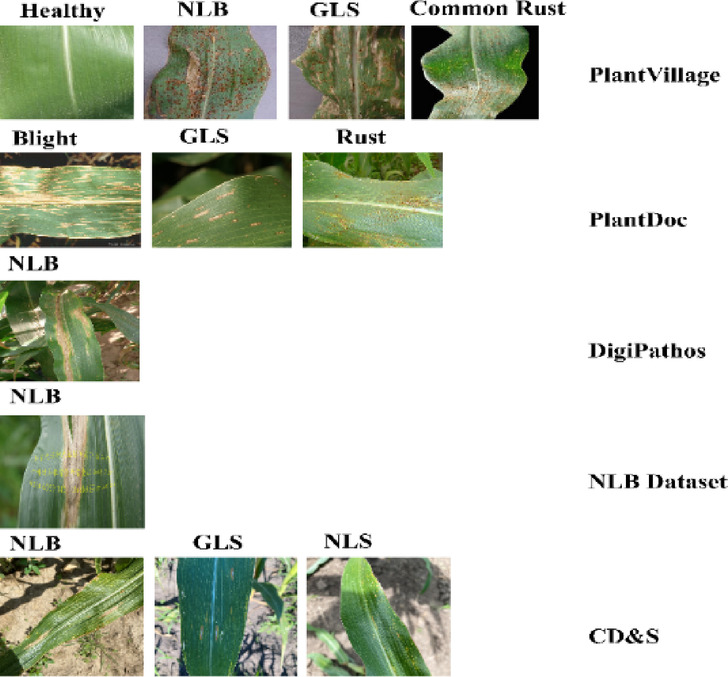
Sample leaf images of corn from publicly available datasets, including PlantVillage, PlantDoc, Digipathos, Northern Leaf Blight (NLB), and Corn Disease & Severity (CD&S).

**Fig. 2:**
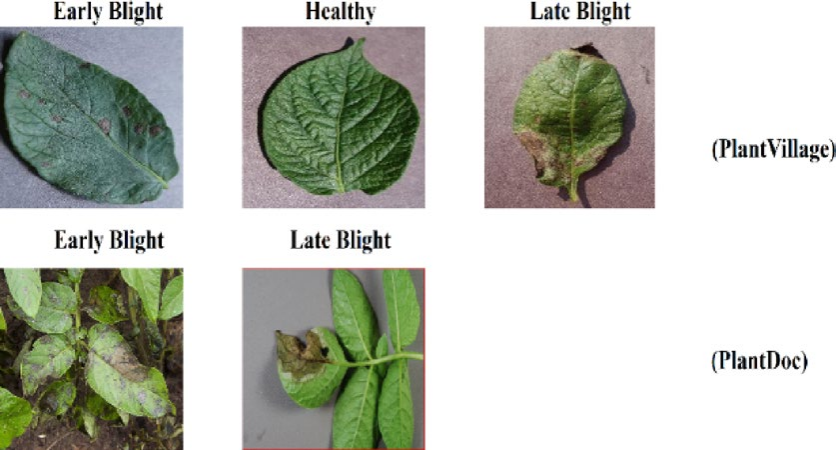
Sample leaf images of potato from publicly available datasets, including PlantVillage and PlantDoc.

This study employed five datasets: PlantVillage, PlantDoc, Northern Leaf Blight (NLB), Digipathos, and Corn Disease & Severity (CD&S) which are publicly available. In the case of corn disease classification, all five datasets mentioned were adopted. Also, even corn leaves images with black background, white background, and without any background included in the CD & S dataset were used. Sample images of all five datasets concerning corn leaves are presented in Fig. 1. In the case of potato disease classification, this study focused only on the potato leaf images that were present in the PlantVillage and PlantDoc datasets. Sample images of Plantvillage and PlantDoc datasets concerning potato leaves are included in Fig. 2.

#### PlantVillage dataset

This study utilized the PlantVillage dataset^26^, which has been famous for quite some time, in the diagnosis of diseases of both corn and potato crops. For corn, a total of 9145 images were selected, from which there were Northern Leaf Blight (NLB), Common Rust, Grey Leaf Spots (GLS), and healthy corn leaves. On the other hand, in the case of potatoes, 2152 images were utilized, comprising three classes: healthy leaves, early blight, and late blight.

#### Plantdoc dataset

In this study, images of corn leaf diseases and potato leaf diseases from the PlantDoc dataset^16^ were taken. As for corn, images of three classes of corn, A. blight (which is known in the current dataset as Northern Leaf Blight or NLB), B. Gray Leaf Spot (GLS), and C. Rust, were taken with 191, 68, and 116 images, respectively. For potatoes, images of early blight and late blight were mined for 222 images.

#### Digipathos dataset

This study utilized 78 Northern Leaf Blight (NLB) images of corn leaves from the Digipathos dataset^20^.

#### Northern leaf blight dataset (nlb)

This study used 116 Northern Leaf Blight (NLB) images of corn leaves from the NLB dataset^21^.

#### Corn disease and severity (cd&s) dataset

The CD&S dataset^29^ includes 497 shots of Northern Leaf Blight (NLB), 523 images of Gray Leaf Spot (GLS), and 551 images of Northern Leaf Spot (NLS). To evaluate model generalization performance, three adjusted datasets with removed backgrounds and uniform black and white backgrounds were employed. While 50% of images were utilized for training.

### Proposed deep learning based CNN model

This study proposed a convolutional neural network (CNN) model that incorporates attention mechanisms. The goal was to increase the model’s ability to focus on essential features in input images, hence improving classification performance while staying computationally economical. The model was designed primarily to handle the challenge of image categorization in resource-constrained environments, ensuring that both model complexity and computing burden are minimized.

#### Model architecture

The proposed CNN model had three convolutional blocks, each followed by a max-pooling technique that gradually lowered the spatial dimensions of the feature maps. The input images (150 × 150 × 3) were first processed using a convolutional layer with 32 3 × 3 filters. This first layer was in charge of capturing low-level information including borders and textures. After adding non-linearity with the ReLU activation function, a max-pooling layer with a pool size of 2 × 2 was employed to down-sample feature maps. This reduced the computational burden in subsequent layers.

The second convolutional block utilized 64 3 × 3 filters to extract complicated patterns from input data. Following this, an attention mechanism was added for the first time to the model, just after the second convolutional block. This attention mechanism was specifically designed to recalibrate feature maps, allowing the network to prioritize the most important channels by assigning varying weights to each one. Following the attention layer, a 2 × 2 max-pooling procedure was applied to further reduce spatial dimensions.

The third convolutional block used 128 3 × 3 filters to extract higher-level information from input images. As with the second block, another attention layer was introduced after the third convolutional block to enhance the feature maps by emphasizing the most significant features at this point of abstraction. This was followed by the final maximum- pooling stage, which had a pool size of 2 × 2.

**Fig. 3:**
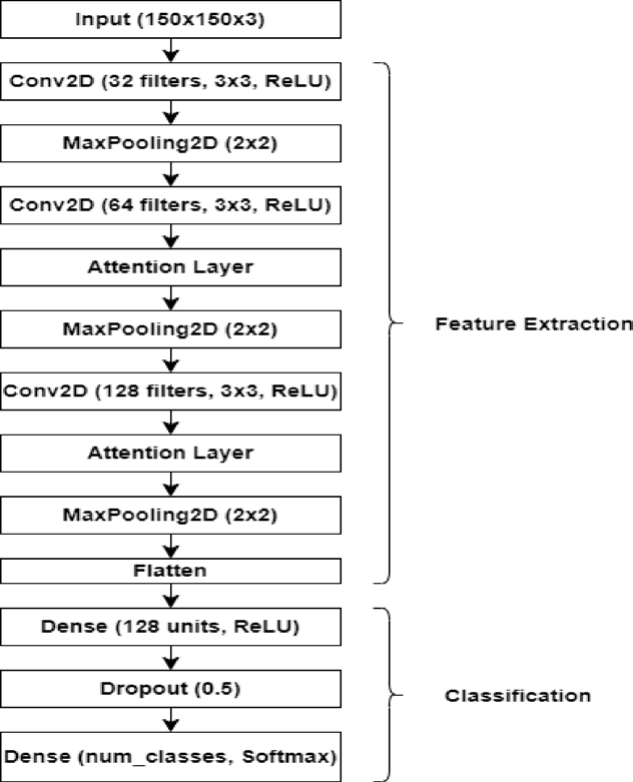
Proposed attention-based CNN architecture for plant disease classification.

Following the convolutional and attention blocks, the resulting feature maps were flattened into a one-dimensional vector. This vector was passed through a dense layer of 128 units, followed by a dropout layer with a 0.5 dropout rate, which was utilized to reduce overfitting by randomly deactivating neurons during training. Finally, the output layer generated class probabilities for multi-class classification using a softmax activation function, with the number of output units equal to the number of classes in the dataset. The proposed Attention-Based CNN model is visually displayed in Fig. 3.

#### Placement of attention layers

The attention layers were carefully positioned after the second and third convolutional blocks to improve feature extraction. The first attention layer, which was added after the second convolutional block, helped the model focus on mid-level elements like textures and forms, which are important for discriminating across classes. By the time the second attention mechanism was activated following the third convolutional block, the network had processed more abstract, high-level features. The attention mechanism at this level kept the model focused on the most relevant features as it progressed further into the network.

This hierarchical application of attention allowed the model to gradually fine-tune its comprehension of the input data, ensuring that both low- and high-level features were appropriately collected and highlighted. By increasing attention to these important points, the model enhanced classification accuracy and generalization capability, even in the face of complex and variable visual data.

#### Hyperparameters

In this study, the proposed attention-based CNN model was applied with the following hyperparameters and configurations. To guarantee consistent input dimensions, each image was reshaped to 150 × 150 pixels. For intra and cross-dataset training, 35%, 15%, and 50% of images were utilized for training, validation, and testing respectively. 50% of images for testing was a good number to check the performance of the proposed model for both the case. The model was trained using a batch size of 32 over 100 epochs. The categorical cross-entropy loss function was used to determine classification error. The model parameters were optimized using the Adam optimizer, which had an initial learning rate of 0.001. These parameters were designed to find a balance between training efficiency and model performance.

#### Dataset augmentation

The ImageDataGenerator class was utilized to improve the training dataset’s robustness and generalizability. The images were rescaled to the [0, 1] range by dividing pixel values by 255. To create variation and simulate various conditions, the augmentation included random rotations of up to 20 degrees, width and height changes of up to 20% of the image dimensions, shear transformations within a 20% range, and zoom alterations of up to 20%. Additionally, horizontal flipping was used to capture a variety of viewpoints. These tactics were designed to diversify the dataset while simultaneously boosting the model’s ability to generalize to new and different inputs.

#### Evaluation metrics

In this study, the generalization performance of the proposed model was assessed by determining testing accuracies for each class using the following formula:

1 $${\mathrm{Testing}}\,{\mathrm{Accuracy}} = \left( {{\mathrm{TP}} + {\mathrm{TN}}} \right)/\left( {{\mathrm{TP}} + {\mathrm{FP}} + {\mathrm{TN}} + {\mathrm{FN}}} \right)$$

True positives are abbreviated as TP, false positives as FP, true negatives as TN, and false negatives as FN. After determining the testing accuracy for each class in the datasets, the results were averaged to provide an overall measure of performance. Additional metrics, such as precision, recall, and F1-score^28^, were used to evaluate the model’s effectiveness. These metrics provided a comprehensive evaluation of the models’ performance across a variety of datasets and categories.

#### Equipment

The experimental work was carried out on a Dell G15 5530 laptop equipped with a 13th Generation Intel® Core™ i5-13450HX processor operating at 2.40 GHz and 16 GB of RAM (15.7 GB usable). The system uses a 64-bit architecture and runs the Windows 11 Home Single Language operating system (Version 23H2, OS Build 22,631.4169). The proposed deep learning model was developed using the Python programming language within the Anaconda Distribution environment, which facilitates the management of scientific libraries and dependencies. Model implementation, experimentation, and result visualization were performed using Jupyter Notebook. The neural network architecture was built using the TensorFlow deep learning framework. To support efficient model training, GPU acceleration was employed using an NVIDIA GeForce RTX 3050 Laptop GPU with 6 GB of dedicated memory. The GPU environment was configured with the NVIDIA CUDA Toolkit and the cuDNN deep neural network library to enable optimized execution of deep learning operations. The software resources used in the study are summarized below, including their respective versions and official access links.

Python (version 3.10): https://www.python.org

Anaconda Distribution (version 2024.02): https://www.anaconda.com

TensorFlow (version 2.10): https://www.tensorflow.org

Jupyter Notebook (version 6.5): https://jupyter.org

NVIDIA CUDA Toolkit (version 11.8): https://developer.nvidia.com/cuda-toolkit

NVIDIA cuDNN (version 8.6): https://developer.nvidia.com/cudnn

### Experimental analysis

#### Intra-dataset training

##### Corn and potato disease classification using different datasets

The proposed model was utilized for corn disease classification using PlantVillage, PlantDoc, and CD & S datasets. Also potato disease classification was done by using PlantVillage and PlantDoc datasets. Testing accuracy, precision, recall, and f1-score were reported in all the six experiments.

##### Binary classification of corn diseases: GLS and NLB, and potato diseases: early blight and late blight

GLS and NLB diseases of corn leaf were general to PlantVillage, PlantDoc, and CD & S datasets. So, the proposed model was used to classify GLS and NLB diseases using the above three datasets. Similarly, early blight and late blight diseases of potatoes were common in PlantVillage and PlantDoc datasets. Hence, the proposed model was utilized to classify these two diseases by using the PlantVillage dataset. PlantDoc was not considered for this because it originally had two classes only, which was already evaluated in the previous case. Testing accuracy, precision, recall, and f1-score from all four experiments were reported.

##### Combined datasets for binary classification

PlantDoc and PlantVillage datasets were combined for GLS and NLB disease classification of corn leaves as well as early blight and late blight disease classification of potato leaves. PlantVillage and CD & S datasets were combined for GLS and NLB disease classification of corn leaves. At last, PlantVillage, PlantDoc, and CD & S datasets were combined for binary GLS and NLB disease classification of corn leaves. Testing accuracy, precision, recall, and f1-score were reported for all four experiments done with the help of the proposed model.

## Result and analysis

### Intra-dataset training

Intra-dataset training is when the model is trained and tested on the images from the same dataset. The following were some experiments conducted for intra-dataset training.

#### Corn and potato disease classification using different datasets

##### PlantVillage dataset for corn disease classification

The corn leaf images of the PlantVillage dataset were utilized for training and evaluating the model.

**Fig. 4:**
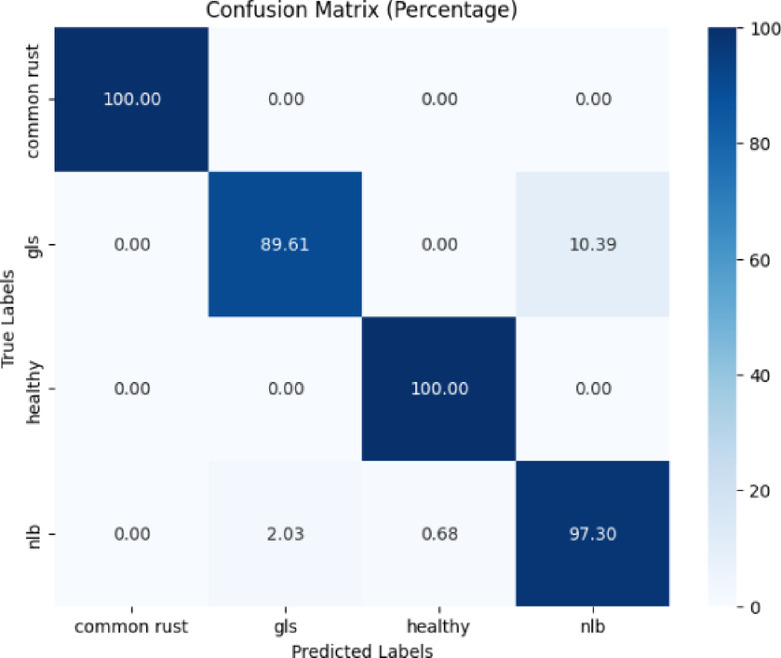
Confusion matrix of the model for corn disease classification using the PlantVillage dataset.

The confusion matrix presented in Fig. 4 revealed that the proposed model was 100% correct for the common rust and healthy classes. Northern leaf blight (NLB) was classified with an accuracy of 97.30%, implying some misclassification. Gray leaf spot (GLS) had the lowest accuracy, at 89.61%.

We obtained various performance metrics like precision, recall, and f1-score for the proposed model. Table 1 presents the classification report of the proposed model. The proposed model achieved 98% testing accuracy for corn disease classification using the PlantVillage dataset, which is an improved performance as compared to the existing studies^13,14,27^, and^15^.

**Table 1:** Classification report of the proposed model for corn disease classification using the plantvillage dataset.

Class	Precision	Recall	F1-score	Accuracy
Common rust	1.00	1.00	1.00	98%
gls	0.96	0.90	0.93	
Healthy	0.99	1.00	1.00	
nlb	0.95	0.97	0.96	

##### PlantVillage dataset for potato disease classification

The proposed model was utilized for Intra-dataset training for potato disease classification using the PlantVillage dataset.

**Fig. 5:**
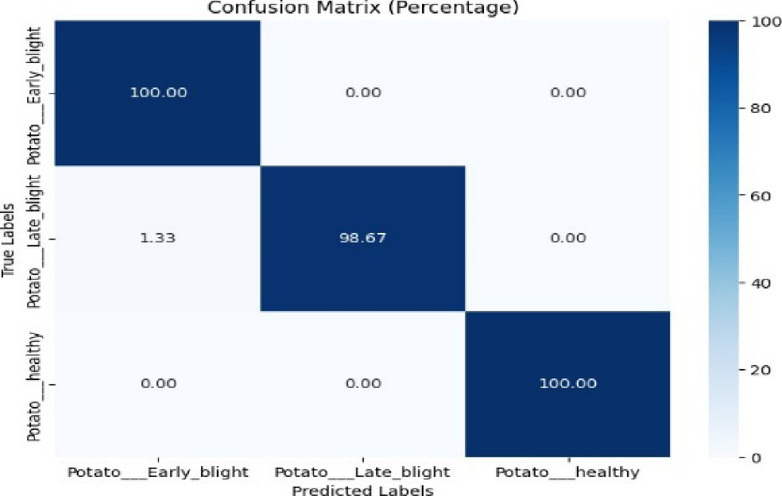
Confusion matrix of the model for potato disease classification using the PlantVillage dataset.

The confusion matrix is displayed in Fig. 5. Figure 5 shows that the proposed model achieved 100% classification accuracy for early blight and healthy class. The model also attained 98.67% classification accuracy for the late blight class.

Table 2 represents the classification report of the proposed model. The proposed model attained 99.38% testing accuracy, which is better than previous studies^17^, and^18^.

**Table 2:** Classification report of the proposed model for potato disease classification using the plantvillage dataset.

Class	Precision	Recall	F1-score	Accuracy
Early blight	0.99	1.00	0.99	99.38%
Late blight	1.00	0.99	0.99	
Healthy	1.00	1.00	1.00	

##### Plantdoc dataset for corn disease classification

The model was utilized for corn disease classification. Images of the PlantDoc dataset were used for training and evaluation.

**Fig. 6:**
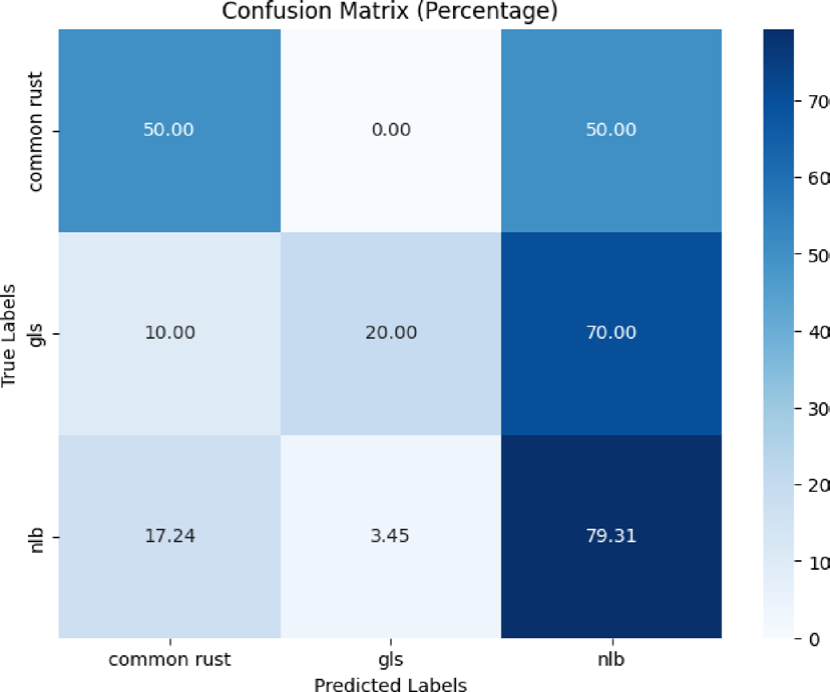
Confusion matrix of the model for corn disease classification using the PlantDoc dataset.

Figure 6 depicts the confusion matrix for the proposed model’s classification of ‘common rust’, ‘gls’wever, 50% were mistakenly classed as ‘nlb’. ‘Gls’ was accurately identified in 20% of cases, whereas 70% were misclassified as ‘nlb’ and 10% were misclassified as ‘gls’. For ‘nlb’, 79.31% were correctly identified, with 17.24% misclassified as ‘common rust’ and 3.45% as ‘gls’.

Several performance metrics were calculated like precision, recall, and f1-score. Table 3 presents the classification report of the proposed model. The proposed model obtained 60% testing accuracy, which is an enhanced result compared to the existing study^16^.

**Table 3:** Classification report of the proposed model for corn disease classification using the plantdoc dataset.

Class	Precision	Recall	F1-score	Accuracy
Common rust	0.60	0.50	0.55	60%
gls	0.67	0.20	0.31	
nlb	0.59	0.79	0.68	

##### Plantdoc dataset for potato disease classification

The proposed model was utilized for training on the leaf images of potato from the PlantDoc dataset and also evaluated using the images of the same dataset.

**Fig. 7:**
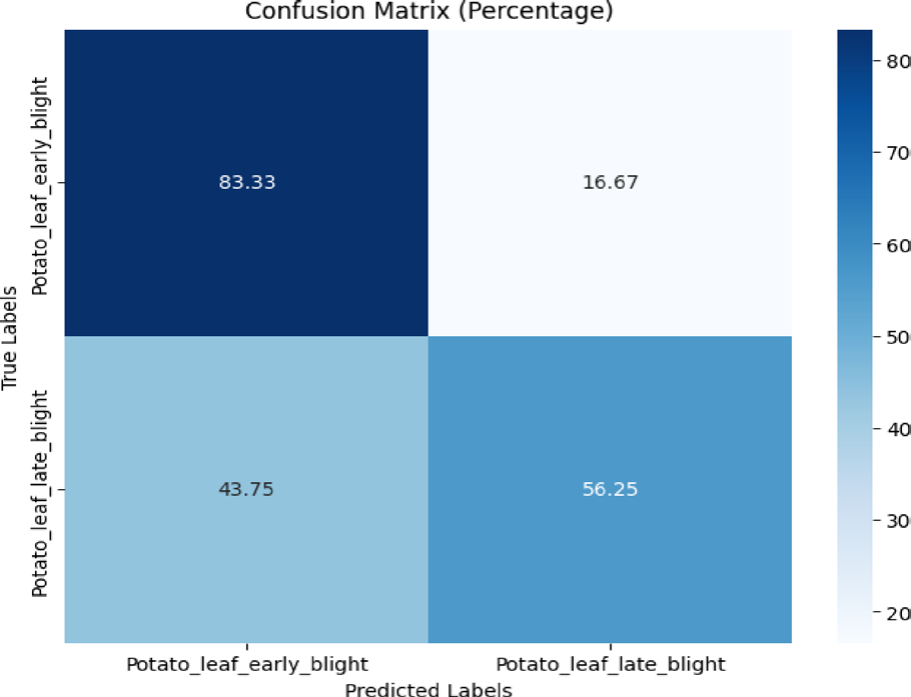
Confusion matrix of the model for potato disease classification using.

The confusion matrix for the proposed model is shown in Fig. 7. Figure 7 reveals that 83.33% of early blight was correctly classified while 16.67% was misclassified as late blight. 56.25% of late blight was correctly classified while 43.75% was misclassified as early blight.

Various performance metrics were calculated like precision, recall, and f1-score. Table 4 displays the classification report of the proposed model. The proposed model achieved 71% testing accuracy in classifying potato diseases.

**Table 4:** Classification report of the proposed model for Potato disease classification using the plantdoc dataset.

Class	Precision	Recall	F1-score	Accuracy
Early blight	0.68	0.83	0.75	71%
Late blight	0.75	0.56	0.64	

##### CD and S dataset for corn disease classification

The model was trained and evaluated using original corn leaf images from the CD & S dataset.

**Fig. 8:**
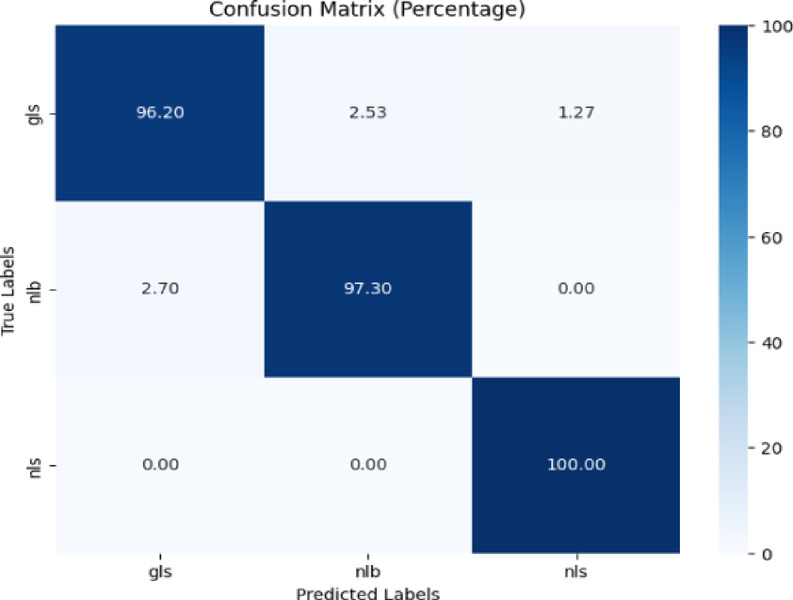
Confusion matrix of the model for corn disease classification using CD & S dataset.

Figure 8 shows the confusion matrix with percentages for three classes: ‘gls’, ‘nlb’, and ‘nls’. The ‘gls’ class predicted 96.20% accurately, with 2.53% mislabeled as ‘nlb’ and 1.27% as ‘nls’. The ‘nlb’ class had 2.70% mislabeled as ‘gls’ yet earned 97.30% accuracy. The ‘nls’ class was predicted correctly with 100% accuracy.

Precision, recall, and f1-score were calculated for the proposed model’s performance. Table 5 presents the classification report of the proposed model. The proposed model attained 98% testing accuracy which is better as compared to the existing study^11^.

**Table 5:** Classification report of the proposed model for corn disease classification using the cd & s dataset.

Class	Precision	Recall	F1-score	Accuracy
gls	0.97	0.96	0.97	98%
nlb	0.97	0.97	0.97	
nls	0.99	1.00	0.99	

##### CD and S removed background images for corn disease classification

The proposed model was trained using the removed background images of the CD & S dataset and evaluated on the images of the same dataset for corn disease classification.

**Fig. 9:**
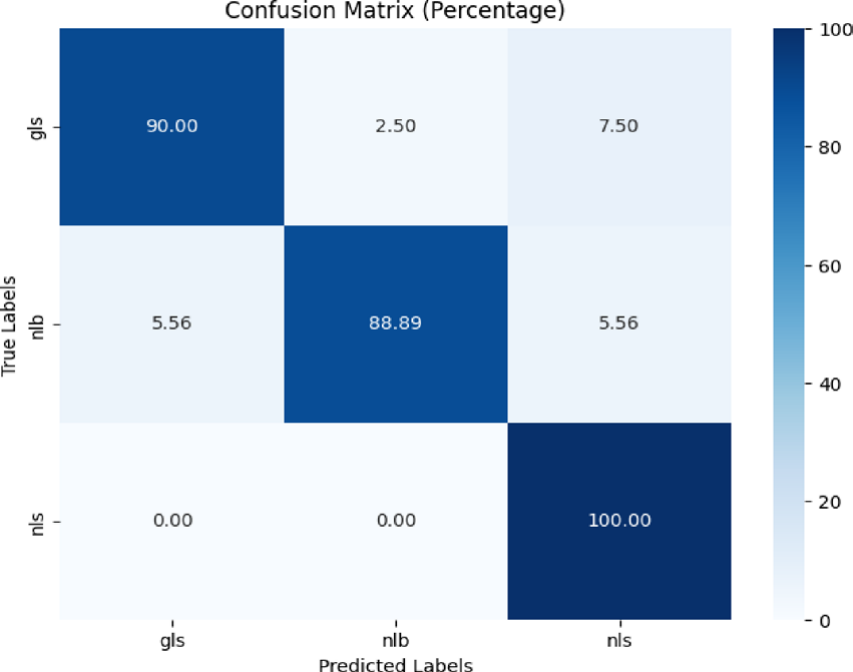
Confusion matrix of the model for corn disease classification using removed background images of CD & S dataset.

Figure 9 shows a confusion matrix in heatmap format, displaying the performance of the proposed model as a percentage across three classes: ‘gls,’ ‘nlb,’ and ‘nls.’ For ‘gls,’ the model predicted 90% correctly, with 2.5% misclassified as ‘nlb’ and 7.5% as ‘nls.’ The ‘nlb’ class was 88.89% correct, with 5.56% misclassified as ‘gls’ and ‘nls.’ The ‘nls’ class achieved flawless accuracy of 100 percent.

Precision, recall, and f1-score were evaluated for the proposed model. Table 6 presents the classification report of the proposed model. Testing accuracy was 93% which is quite high as compared to the existing study^12^.

**Table 6:** Classification report of the proposed model for corn disease classification using removed background images of the cd & s dataset.

Class	Precision	Recall	F1-score	Accuracy
gls	0.95	0.90	0.92	93%
nlb	0.97	0.89	0.93	
nls	0.89	1.00	0.94	

#### Binary classification of corn diseases: gls and nlb, and potato diseases: early blight and late blighT

GLS and NLB diseases were general to the PlantVillage, PlantDoc, and CD & S datasets for corn images. Also, early blight and late blight diseases were common in PlantVillage and PlantDoc datasets for potato leaf images. So, therefore these binary classifications were done to explore more about the proposed model.

##### PlantVillage dataset for GLS and NLB classification of corn leaves

The proposed model was used to train on the images of corn leaves from the PlantVillage dataset from where only gls and nlb disease images were taken. The model was evaluated on the images of same dataset excluding the healthy class images.

**Fig. 10:**
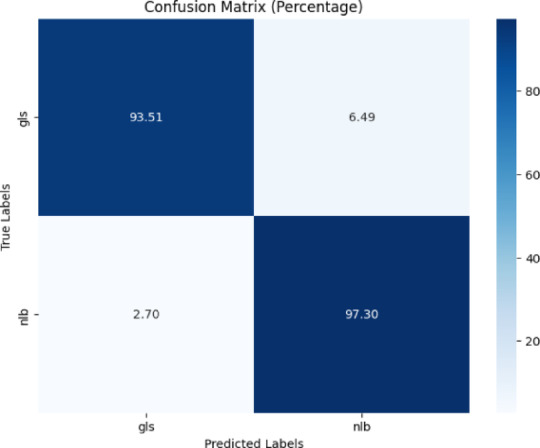
Confusion matrix of the model for gls, and nlb disease classification of corn leaves using gls and nlb images of the PlantVillage dataset.

Figure 10 represented the confusion matrix as a heatmap, with percentage values for two classes: ‘gls’ and ‘nlb.’ The top left quadrant showed 93.51% true positives for ‘gls,’ whereas the top right showed 6.49% false positives. The bottom left exhibited 2.70% false negatives for ‘gls,’ whereas the bottom right showed 97.30% true positives for ‘nlb.’

Several performance metrics were evaluated like precision, recall, and f1-score. Table 7 displays the classification report of the model. Testing accuracy was reported as 96% for gls, and nlb disease classification.

**Table 7:** Classification report of the proposed model for gls, and nlb disease classification using the plantvillage dataset.

Class	Precision	Recall	F1-score	Accuracy
gls	0.95	0.94	0.94	96%
nlb	0.97	0.97	0.97	

##### PlantVillage dataset for early blight and late blight disease classification of potato leaves

The proposed model was trained on the images of early blight, and late blight diseases and tested on the images of early blight, and late blight of the PlantVillage dataset.

**Fig. 11:**
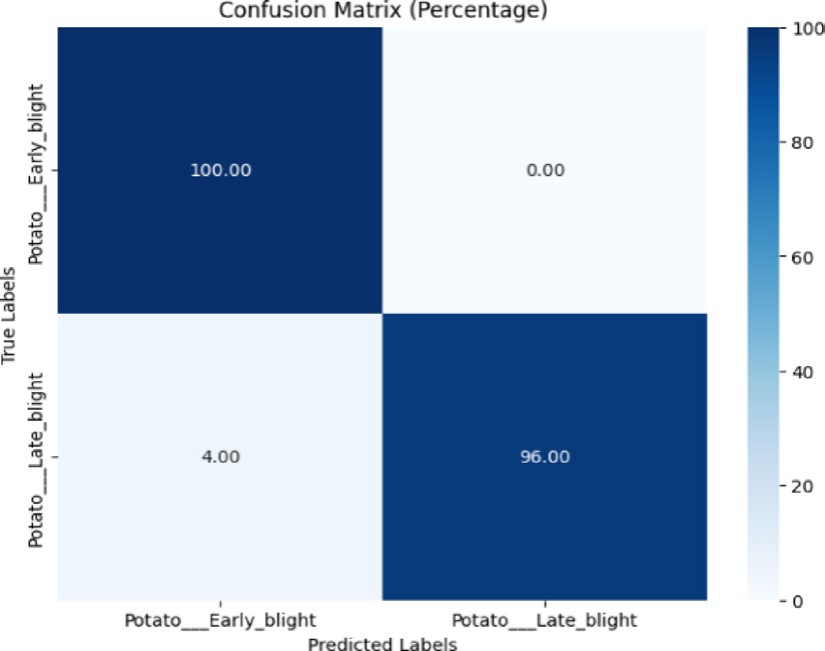
Confusion matrix of the model for early blight, and late blight disease classification of potato leaves using early blight and late blight images of the PlantVillage dataset.

The confusion matrix for the proposed model is displayed in Fig. 11. Figure 11 showed perfect classification for ‘Potato Early Blight,’ whereas ‘Potato Late Blight’ achieved 96% accuracy, with only 4% misclassified as ‘Early Blight,’ suggesting effective disease distinction.

Table 8 represents the classification report of the proposed model. The testing accuracy was 98%.

**Table 8:** Classification report of the proposed model for early blight, and late blight disease classification using the plantvillage dataset.

Class	Precision	Recall	F1-score	Accuracy
Early blight	0.96	1.00	0.98	98%
Late blight	1.00	0.96	0.98	

##### Plantdoc dataset for GLS, and NLB disease classification of corn leaves

The proposed model had considered gls, and nlb images of the Plantdoc dataset for classifying the gls, and nlb diseases of corn leaves. Then it was evaluated on the gls, and nlb images of the same dataset.

**Fig. 12:**
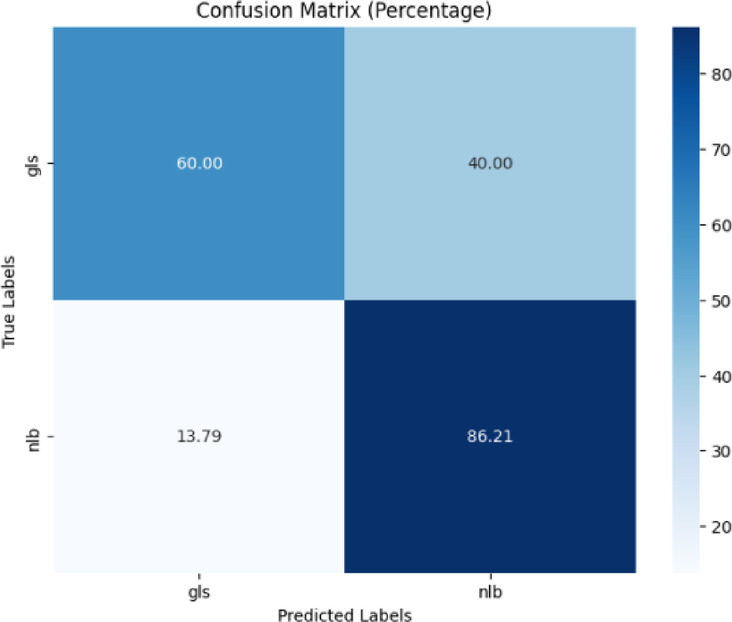
Confusion matrix of the model for gls, and nlb disease classification of corn leaves using gls and nlb images of the PlantDoc dataset.

Figure 12 depicted a confusion matrix in percentage format, which demonstrated the performance of the proposed model. The top-left cell showed that 60% of ‘gls’ were accurately predicted as ‘gls,’ while the top-right cell showed that 40% of ‘gls’ were incorrectly categorized as ‘nlb.’ The bottom-left cell revealed that 13.79% of ‘nlb’ were wrongly classified as ‘gls,’ while the bottom-right cell showed that 86.21% of ‘nlb’ were correctly forecasted as ‘nlb.’

The classification report of the proposed model is displayed in Table 9. Precision, recall, and f1-score were displayed in Table 9. The testing accuracy was 82% for gls, and nlb classification using the PlantDoc dataset.

**Table 9:** Classification report of the proposed model for gls, and nlb disease classification using the plantdoc dataset.

Class	Precision	Recall	F1-score	Accuracy
gls	0.60	0.60	0.60	82%
nlb	0.86	0.86	0.86	

##### CD and S dataset for GLS, and NLB disease classification of corn leaves

Original corn leaf images were utilized for training and evaluating the model. Only gls, and nlb images were considered.

**Fig. 13:**
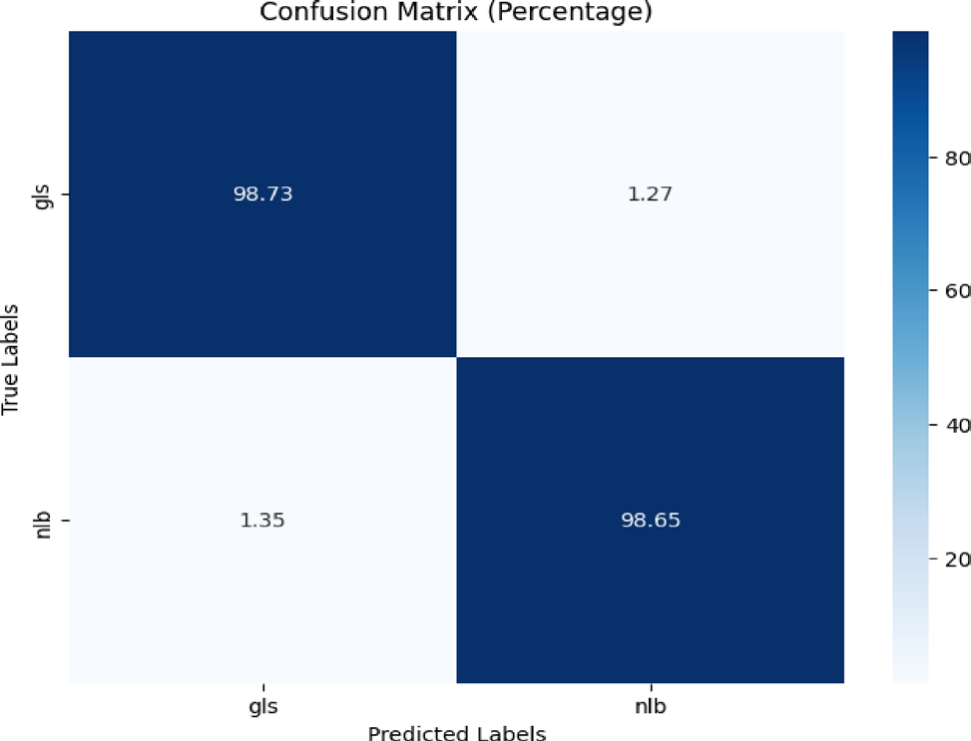
Confusion matrix of the model for gls, and nlb disease classification of corn leaves using gls and nlb images of the CD & S dataset.

The confusion matrix of the proposed model is displayed in Fig. 13 which showed a true positive rate of 98.73% for ‘gls,’ with 1.27% mistakenly categorized as ‘nlb.’ The rate was 98.65% for ‘nlb,’ with 1.35% misclassified as ‘gls.’

The classification report is displayed in Table 10 for the proposed model for gls, and nlb classification using CD & S dataset. The testing accuracy was quite high i.e. 99%.

**Table 10:** Classification report of the proposed model for gls, and nlb disease classification using the cd & s dataset.

Class	Precision	Recall	F1-score	Accuracy
gls	0.99	0.99	0.99	99%
nlb	0.99	0.99	0.99	

#### Combined datasets for binary classification

##### Combined PlantVillage and plantdoc dataset for GLS and NLB disease classification for corn

The PlantVillage and PlantDoc datasets were combined by taking images of gls, and nlb from both the datasets. The proposed model was utilized for training on the images of gls, and nlb from the combined dataset and also tested on the images of gls, and nlb from the combined dataset.

**Fig. 14:**
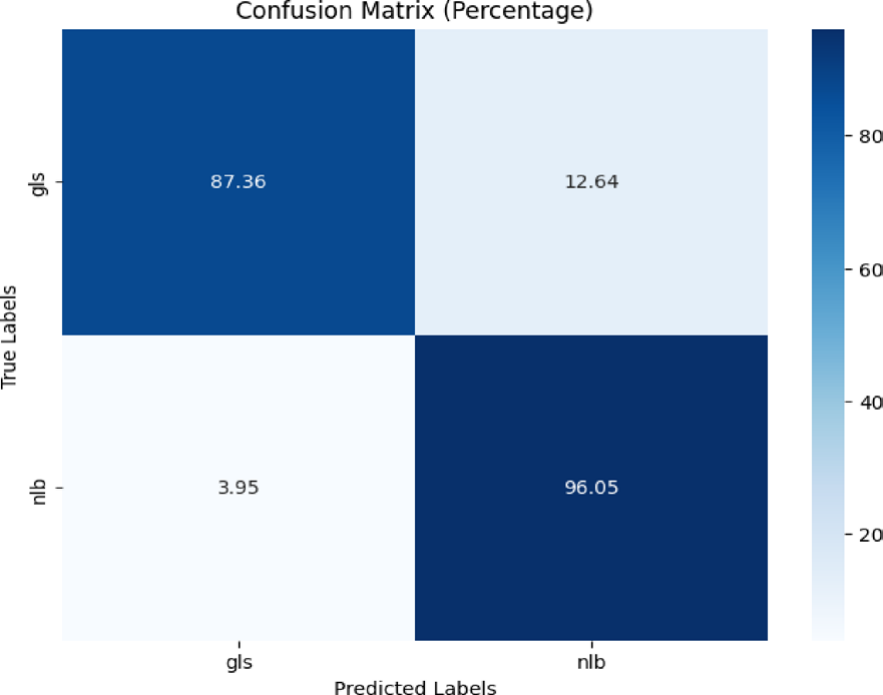
Confusion matrix of the model for gls, and nlb disease classification of corn leaves using gls and nlb images of the combined plantvillage and plantdoc dataset.

The confusion matrix is displayed in Fig. 14. Figure 14 revealed 87.36% correct predictions for ‘gls’ and 96.05% for ‘nlb,’ with 12.64% misclassifications of ‘gls’ as ‘nlb’ and 3.95% of ‘nlb’ as ‘gls.’

Table 11 presents the classification report of the model. The testing accuracy was 93%.

**Table 11:** Classification report of the proposed model for gls, and nlb disease classification using the combined plantvillage and plantdoc dataset.

Class	Precision	Recall	F1-score	Accuracy
gls	0.92	0.87	0.89	93%
nlb	0.94	0.96	0.95	

##### Combined plantvillage and plantdoc dataset for early blight and late blight classification for potato leaves

The PlantVillage and PlantDoc datasets were combined by taking images of early blight, and late blight from both the datasets. The proposed model was utilized for training on the images of early blight, and late blight from the combined dataset and also tested on the images of early blight, and late blight from the combined dataset.

**Fig. 15:**
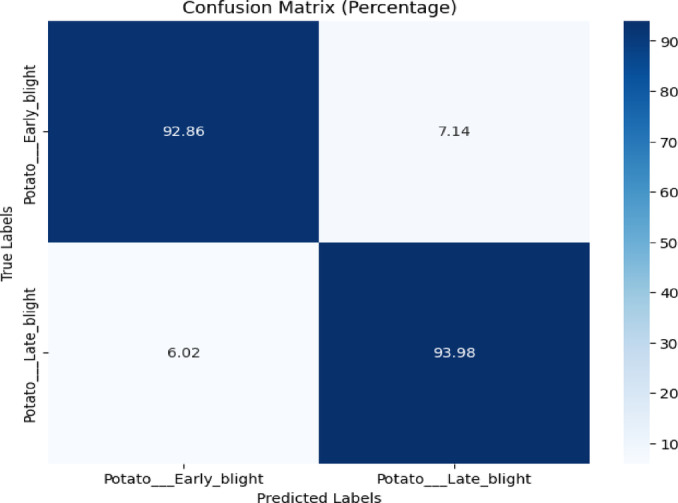
Confusion matrix of the model for early blight, and late blight disease classification of potato leaves using early blight and late blight images of the combined plantvillage and plantdoc dataset.

The confusion matrix displayed in Fig. 15 revealed that ‘Potato Early Blight’ achieved 92.86% accuracy, while ‘Potato Late Blight’ reached 93.98%, with misclassification rates of 7.14% and 6.02%, respectively.

Table 12 displays the classification report of the model for early blight and late blight classification. Testing accuracy was 93.41%.

**Table 12:** Classification report of the proposed model for early blight and late blight disease classification using the combined plantvillage and plantdoc dataset.

Class	Precision	Recall	F1-score	Accuracy
Early blight	0.94	0.93	0.93	93.41%
Late blight	0.93	0.94	0.93	

##### Combined plantvillage and CD and S dataset for GLS, and NLB disease classification for corn leaves

The PlantVillage and CD & S datasets were combined by taking images of gls, and nlb from both the datasets. The proposed model was utilized for training on the images of gls, and nlb from the combined dataset and also tested on the images of gls, and nlb from the combined dataset.

**Fig. 16:**
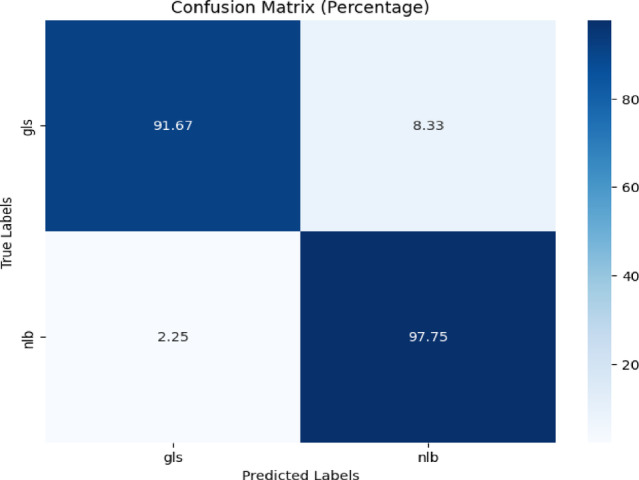
Confusion matrix of the model for gls, and nlb disease classification of corn leaves using gls and nlb images of the combined plantvillage and CD & S dataset.

The confusion matrix depicted in Fig. 16 showed 91.67% accuracy for ‘gls’ and 97.75% for ‘nlb,’ with misclassification rates of 8.33% and 2.25%, respectively.

Table 13 displays the classification report of the proposed model. The testing accuracy was 94%.

**Table 13:** Classification report of the proposed model for gls and nlb disease classification using the combined plantvillage and cd & s dataset.

Class	Precision	Recall	F1-score	Accuracy
gls	0.97	0.92	0.94	94%
nlb	0.94	0.98	0.96	

##### Combined plantvillage, plantdoc, and CD and S dataset for GLS, and NLB disease classification of corn leaves

The PlantVillage, PlantDoc and CD & S datasets were combined by taking images of gls, and nlb from all three of the datasets. The proposed model was utilized for training on the images of gls, and nlb from the combined dataset and also tested on the images of gls, and nlb from the combined dataset.

**Fig. 17:**
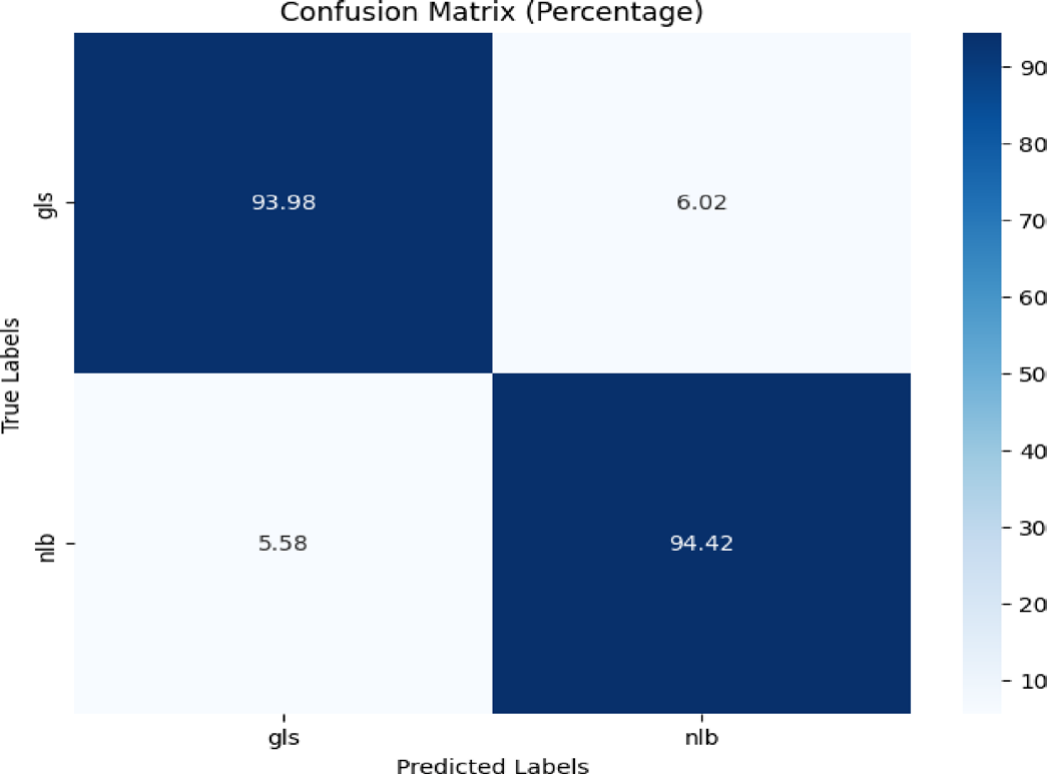
Confusion matrix of the model for gls, and nlb disease classification of corn leaves using gls and nlb images of the combined plantvillage, plantdoc, and CD & S dataset.

The confusion matrix is depicted in Fig. 17. Figure 17 demonstrated 93.98% accuracy for ‘gls’ and 94.42% for ‘nlb,’ with corresponding misclassification rates of 6.02% and 5.58%.

Table 14 depicts the classification report of the proposed model for gls, and nlb disease classification using combined PlantVillage, PlantDoc, and CD & S dataset. The testing accuracy was 94%.

**Table 14:** Classification report of the proposed model for gls and nlb disease classification using the combined plantvillage, plantdoc and cd & s dataset.

Class	Precision	Recall	F1-score	Accuracy
gls	0.92	0.94	0.93	94%
nlb	0.96	0.94	0.95	

Table 15 displays the comparison between the existing studies and the proposed model for intra- dataset training. For intra-dataset training, the proposed model performed best for potato disease classification using the PlantVillage dataset by attaining 99.38% accuracy.

**Table 15:** Comparison table of existing study and the proposed model for intra-dataset training.

Study	Dataset	Accuracy
^27^	PlantVillage (Corn)	96.76%
^13^	PlantVillage (Corn)	93.44%
^14^	PlantVillage (Corn)	97.82%
^15^	PlantVillage (Corn)	94.52%
^12^	PlantVillage (Potato)	98.12%
^17^	PlantVillage (Potato)	95.37%
^16^	PlantDoc (Corn)	57%
^11^	CD & S (Corn)	97.89%
^12^	CD & S No Background (Corn)	87.52%
Proposed model	PlantVillage (Corn)	98%
	PlantDoc(Corn)	60%
	CD & S(Corn)	98%
	CD & S No Background (Corn)	93%
	PlantVillage (Potato)	99.38%
	PlantDoc (Potato)	71%
	PlantVillage (Gls, and Nlb of Corn)	96%
	PlantDoc (Gls, and Nlb of Corn)	82%
	CD & S (Gls, and Nlb of Corn)	99%
	PlantVillage (Early blight and late blight of potato)	98%
	PlantVillage + PlantDoc (Gls, and Nlb of Corn)	93%
	PlantVillage + PlantDoc (Early blight, and Late Blight of Potato)	93.41%
	PlantVillage + CD & S (Gls, and Nlb of Corn)	94%
	PlantVillage + CD & S + PlantDoc (Gls, and Nlb of Corn)	94%

### Cross-dataset training

Cross-dataset training means training on one dataset and testing on another dataset. The proposed model attained the highest average classification accuracy for corn disease classification when it was utilized for training on the no- background images of the CD & S dataset and tested on the leaf images of other datasets which is explained with all the results in detail. Potato leaf diseases were also classified by doing the training on the PlantVillage dataset and testing on the PlantDoc dataset. The vice versa was also done in this study. Potato disease classification using cross-dataset training is explained in detail with all the results. We depicted all our results related to cross-dataset training for corn disease classification and potato disease classification in Tables 23 and 24 respectively. Following are some experiments conducted for cross-dataset training in this study.

#### CD and S dataset having no background (corn)

The model was trained using images without backgrounds from the CD and S dataset.

##### PlantVillage dataset

The proposed model, which was trained on background- removed pictures from the CD & S dataset, was tested using corn leaf pictures from the PlantVillage dataset. GLS & NLB classes were general in both datasets, so they were considered for the evaluation.

**Fig. 18:**
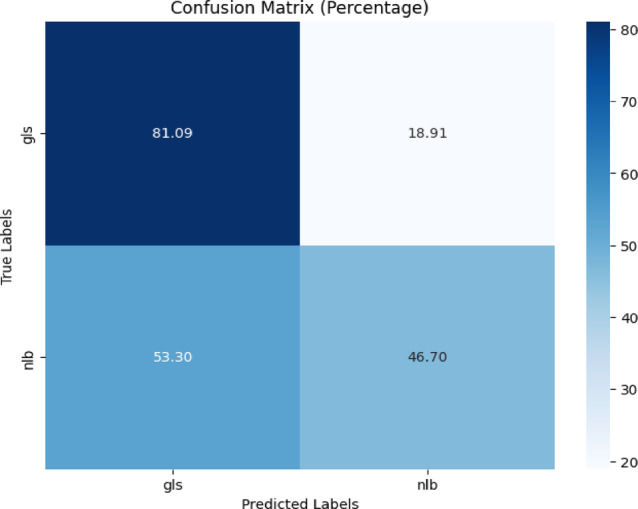
Confusion matrix of the model for corn disease classification using gls, and nlb images from the PlantVillage dataset.

The confusion matrix is depicted in Fig. 18. Figure 18 revealed that the model correctly detected 81.09% of the ‘gls’ instances, but incorrectly classified 18.91%. It correctly predicted 46.70% of ‘nlb’ cases, but incorrectly classified 53.30% as ‘gls’. Table 16 gives the classification report of the proposed model for corn disease classification from the PLANTVILLAGE dataset.

**Table 16:** Classification report of the proposed model for corn disease classification from plantvillage dataset.

Class	Precision	Recall	F1-score	Accuracy
gls	0.44	0.81	0.57	81.09%
nlb	0.83	0.47	0.60	46.70%

##### Plantdoc dataset

**Fig. 19:**
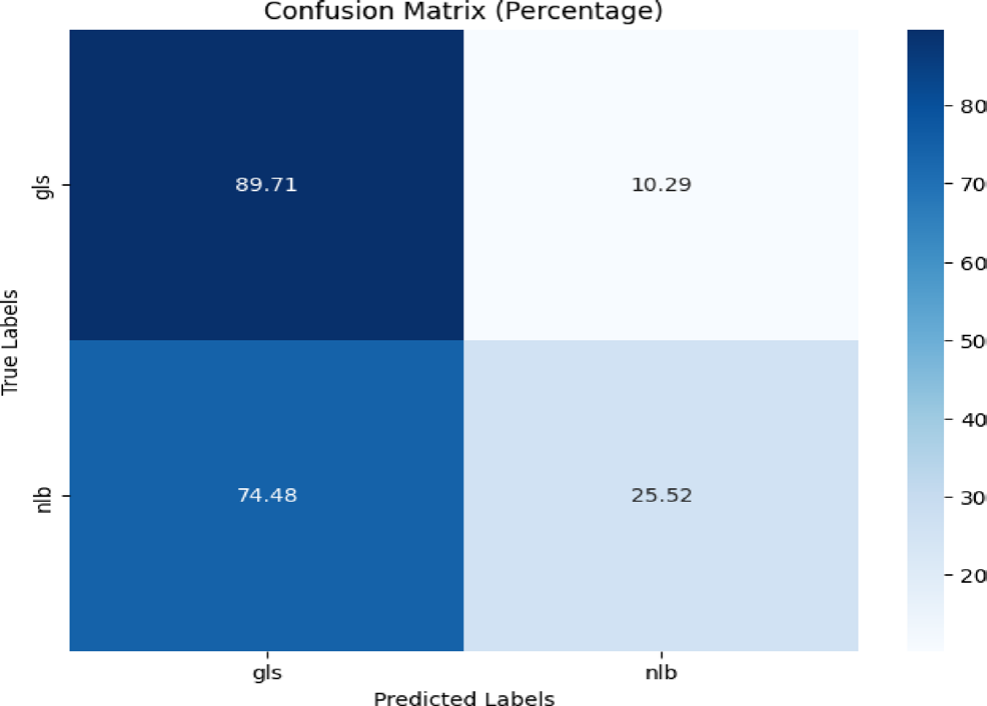
Confusion matrix of the model for corn disease classification using gls, and nlb images from the PlantDoc dataset.

The model was trained using background-free images from the CD & S dataset and tested with images from the PlantDoc dataset. GLS & NLB diseases were common in both datasets, so they were considered for the evaluation. The confusion matrix and classification reports are depicted in Fig. 19 and Table 17, respectively.

**Table 17:** Classification report of the proposed model for corn disease classification from plantdoc dataset.

Class	Precision	Recall	F1-score	Accuracy
gls	0.29	0.76	0.42	89.71%
nlb	0.80	0.33	0.47	25.52%

Figure 19 showed that the proposed model classified 89.71% of gls and 25.52% of nlb correctly. 74.48% of the nlb were misclassified as gls.

##### Digipathos dataset

**Fig. 20:**
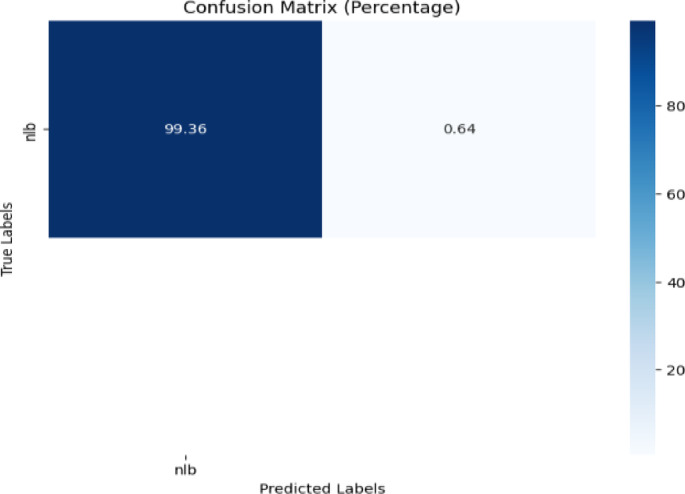
Confusion matrix of the model for corn disease classification using nlb images from the Digipathos dataset.

NLB class was common between CD & S and Digipathos dataset. So, no background images of gls, and nlb of CD & S datasets were used for training. The confusion matrix is needed which is displayed in Fig. 20.

Figure 20 depicts that the model performed magnificently by achieving 99.36% accuracy for nlb disease classification.

##### NLB dataset

**Fig. 21:**
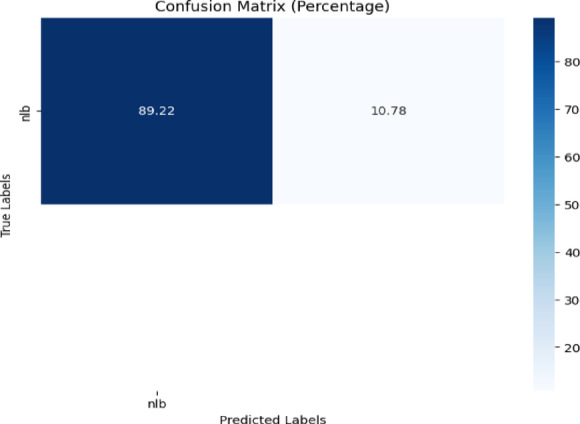
Confusion matrix of the model for corn disease classification using nlb images from the NLB dataset.

In this case, the NLB class is general in both the CD & S dataset and the NLB dataset. As a result, as with the previous experiment, background-free images of GLS and NLB from the CD & S dataset were used to train the model. Then the model was evaluated on the nlb images from the NLB dataset. A confusion matrix is presented in Fig. 21 to show the performance of the model.

Figure 21 revealed that the proposed model achieved 89.22% accuracy for the classification of nlb diseases.

##### CD and S dataset with original images

The images having no background from the CD & S dataset were considered for training and original images of corn leaf were utilized for evaluating the model. The three classes used were gls, nlb, and nls respectively.

**Fig. 22:**
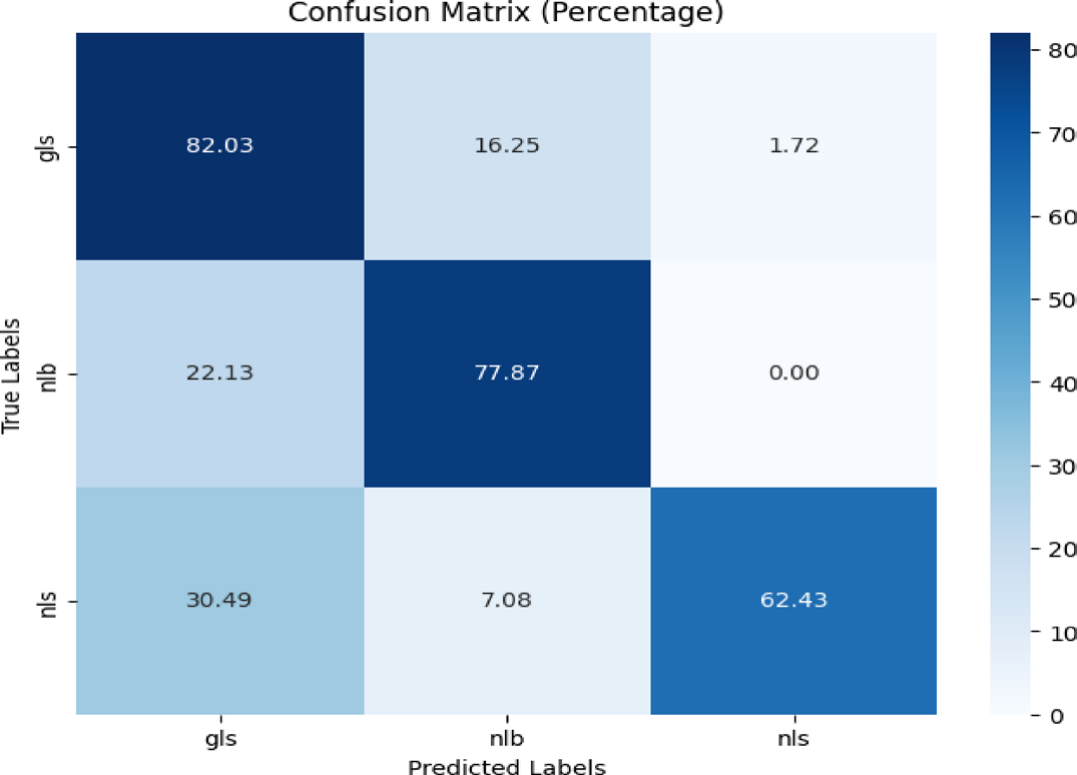
Confusion matrix of the model for corn disease classification using original images from CD & S dataset.

The confusion matrix is presented in Fig. 22 which revealed that the proposed model achieved 82.03%, 77.87%, and 62.43% accuracy for gls, nlb, and nls disease classification respectively. Table 18 gives the classification report of the proposed model for corn disease classification using original images from the CD & S dataset.

**Table 18:** Classification report of the proposed model for corn disease classification using original images from the cd & s dataset.

Class	Precision	Recall	F1-score	Accuracy
gls	0.61	0.82	0.70	82.03%
nlb	0.76	0.78	0.77	77.87%
nls	0.97	0.62	0.76	62.43%

##### CD and S dataset with black background images

**Fig. 23:**
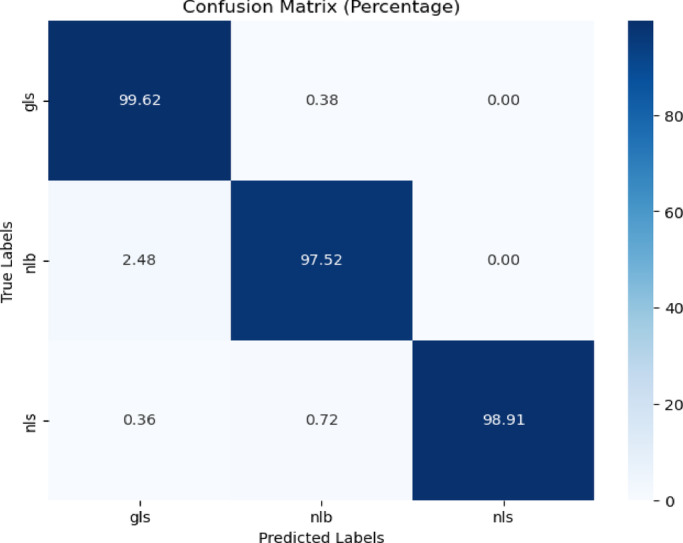
Confusion matrix of the model for corn disease classification using black background images from CD & S dataset.

The proposed model was trained on the no background images of the CD &S dataset just like the previous case and tested on the black background images of gls, nlb, and nls . The confusion matrix and classification report are represented in Fig. 23 and Table 19 respectively.

**Table 19:** Classification report of the proposed model for corn disease classification using black backgroung images from the cd & s dataset.

Class	Precision	Recall	F1-score	Accuracy
gls	0.97	1.00	0.98	99.62%
nlb	0.99	0.98	0.98	97.52%
nls	1.00	0.99	0.99	98.91%

Figure 23 revealed that the model performed superbly by achieving 99.62%, 97.52%, and 98.91% accuracy for the classification of gls, nlb, and nls diseases.

##### CD and S dataset with white background images

**Fig. 24:**
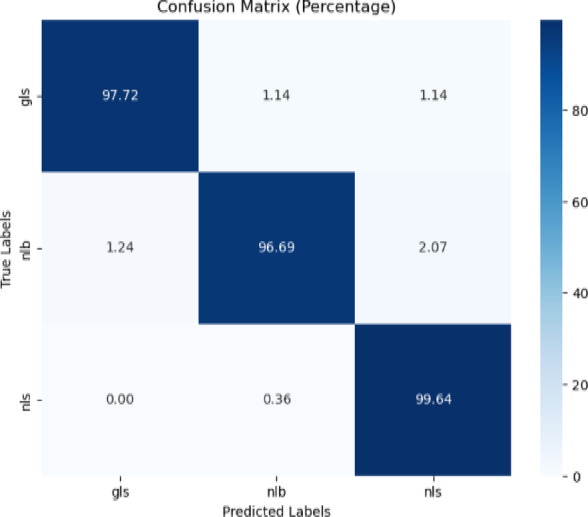
Confusion matrix of the model for corn disease classification using white background images from CD & S dataset.

The proposed model was trained just like the previous two cases and evaluated on the white background images of CD & S dataset. The confusion matrix and classification report are displayed in Fig. 24 and Table 20 respectively.

**Table 20:** Classification report of the proposed model for corn disease classification using white background images from the cd & s dataset.

Class	Precision	Recall	F1-score	Accuracy
gls	0.99	0.98	0.98	97.72%
nlb	0.98	0.97	0.97	96.69%
nls	0.97	1.00	0.98	99.64%

Figure 24 revealed that the proposed model was very good for the classification of gls, nlb, and nls by achieving 97.72%, 96.69%, and 99.64% accuracy respectively.

#### PlantVillage dataset (training) and PLANTDOC dataset (testing) for potato

Early blight and late blight were two common classes in PlantVillage and PlantDoc dataset of Potato. The proposed model was trained using the images of Plantvillage dataset and checked on the images of Plantdoc dataset.

**Fig. 25:**
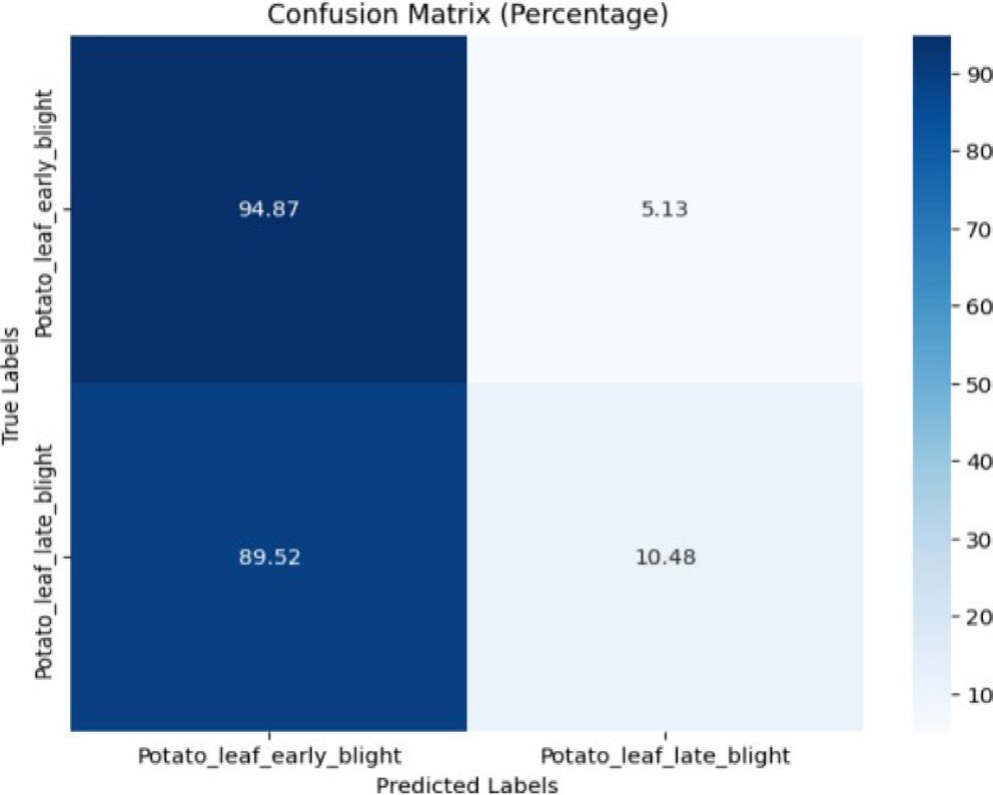
Confusion matrix of the model for corn disease classification using early blight and late blight images from the PlantDoc dataset.

The confusion in Fig. 25 depicted that the proposed model achieved 94.87% accuracy for early blight while only 10.48% of late blight images were correctly classified. Table 21 gives the classification report of the proposed model for potato disease classification from PLANTDOC dataset.

**Table 21:** Classification report of the proposed model for potato disease classification from plantdoc dataset.

Class	Precision	Recall	F1-score	Accuracy
Early blight	0.54	0.95	0.69	94.87%
Late blight	0.65	0.10	0.18	10.48%

#### PlantDoc dataset (training) and PlantVillage dataset (testing) for potato

**Fig. 26:**
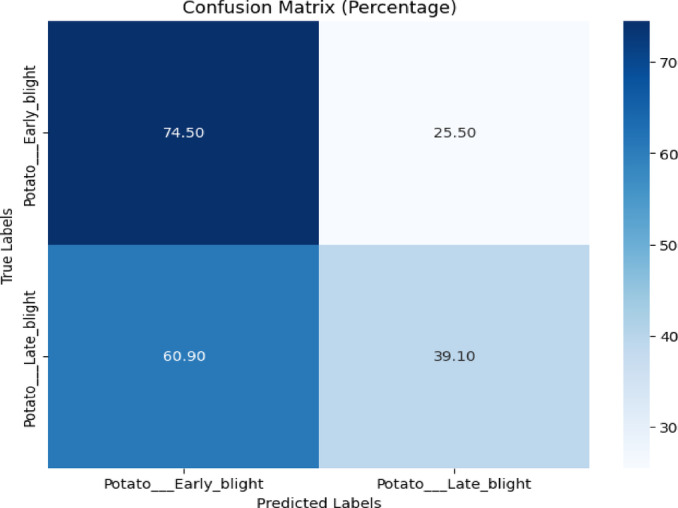
Confusion matrix of the model for potato disease classification using early blight and late blight images from the PlantVillage dataset.

The proposed model was utilized for training on the images of the PlantDoc dataset and testing on the images of the PlantVillage dataset. The confusion matrix and Classification report are depicted in Fig. 26 and Table 22 respectively.

**Table 22:** Classification report of the proposed model for potato disease classification from plantvillage dataset.

Class	Precision	Recall	F1-score	Accuracy
Early blight	0.55	0.74	0.63	74.50%
Late blight	0.61	0.39	0.48	39.10%

Figure 26 revealed that the proposed model achieved 74.50% and 39.10% in classifying early blight and late blight, respectively.

#### CD & S dataset with original images (corn)

##### Plantvillage dataset

Original corn leaf images of the CD & S dataset were used to train the proposed model and images of the PlantVillage dataset were utilized to evaluate the proposed model. Only gls, and nlb images were considered due to the common classes in both the dataset. The proposed model attained 84% of accuracy for gls but only 4% for nlb.

##### Plantdoc dataset

Original corn leaf images of the CD & S dataset were used to train the proposed model and images of the PlantDoc dataset were utilized to evaluate the proposed model. Here also only gls, and nlb images were taken. The proposed model achieved 56% and 66% accuracy in classifying gls, and nlb diseases respectively.

##### Digipathos dataset

Original corn leaf images of the CD & S dataset were used to train the proposed model and images of the Digipathos dataset were utilized to evaluate the proposed model. Here also only gls, and nlb images were taken for training but only nlb images were considered from the Digipathos dataset for testing. 58% of nlb images were accurately classified by the proposed model.

##### NLB dataset

Original corn leaf images of the CD & S dataset were used to train the proposed model and images of the NLB dataset were utilized to evaluate the proposed model. Here also only gls, and nlb images were considered for training but only nlb images were considered from the NLB dataset for evaluation. The proposed model performed magnificently by achieving 94% of classification accuracy for nlb images.

##### CD and S dataset with black background images

Original corn leaf images of the CD & S dataset were used to train the proposed model and images having black background of the CD & S dataset were utilized to evaluate the proposed model. Gls, nlb, and NLS images were considered for training and testing. 69%, 63%, and 98% of gls, nlb, and nls respectively were correctly classified by the model.

##### CD and S dataset with white background images

Original corn leaf images of the CD & S dataset were used to train the proposed model and images having white background of the CD & S dataset were utilized to evaluate the proposed model. Gls, nlb, and NLS images were considered for training and evaluation. The proposed model was able to correctly classify 97% of gls, 72% of nlb, and 56% of nls.

#### PlantVillage dataset (corn)

##### PlantDoc dataset

The PlantVillage dataset was considered for training the proposed model by taking gls, and nlb images and evaluated on the PlantDoc dataset’s gls, and nlb images. The proposed model was able to achieve 6.03%, 79.41%, and 19.27% classification accuracy for gls, nlb and rust diseases respectively.

##### Digipathos dataset

The PlantVillage dataset was considered for training the proposed model by taking gls, and nlb images and evaluated on the Digipathos dataset’s nlb images only. 40.25% of nlb diseases were correctly classified by the model.

##### NLB Dataset

The PlantVillage dataset was considered for training the proposed model by taking gls, and nlb images and evaluated on the NLB dataset’s nlb images only. The model attained 17.25% of accuracy for nlb diseases.

##### CD and S with original images

The PlantVillage dataset was utilized for training the proposed model by taking gls, and nlb images and evaluated on the CD & S dataset’s gls, and nlb images. 15.25% of gls, and 99.60% of nlb were accurately classified by the model.

##### CD and S with black background images

The PlantVillage dataset was utilized for training the proposed model by taking gls, and nlb images and evaluated on the CD & S dataset’s black background images of gls, and nlb. 100% accuracy was achieved in classifying nlb diseases but only 20.85% for gls diseases.

##### CD and S with white background images

The PlantVillage dataset was utilized for training the proposed model by taking gls, and nlb images and evaluated on the CD & S dataset’s white background images of gls, and nlb. The proposed model was able to classify 96.69% of nlb images correctly while only 24.25% of gls images were correctly classified.

#### PlantDoc dataset (corn)

##### PlantVillage dataset

The PlantDoc dataset was used for training the proposed model by taking gls, and nlb images and evaluated on the PlantVillage dataset’s gls, and nlb images. The model achieved highest 88.44% accuracy for categorizing nlb images, while 8.58% and 35.40% for gls and rust images respectively.

##### Digipathos dataset

The PlantDoc dataset was used for training the proposed model by taking gls, and nlb images and evaluated on the Digipathos dataset’s nlb images only. Only 21.55% of nlb images were correctly categorized.

##### NLB Dataset

The PlantDoc dataset was used for training the proposed model by taking gls, and nlb images and evaluated on the NLB dataset’s nlb images only. The proposed model only achieved 22.30% of classification accuracy for nlb images.

##### CD and S with original images

The PlantDoc dataset was utilized for training the proposed model by taking gls, and nlb images and evaluated on the CD & S dataset’s gls, and nlb images. 99.40% of nlb images were rightly classified, but only 5.25% of gls were correctly classified.

##### CD and S with black background images

The PlantDoc dataset was utilized for training the proposed model by taking gls, and nlb images and evaluated on the CD & S dataset’s black background images of gls, and nlb. The proposed model was able to classify 97.93% of nlb images accurately but only 5.46% of gls images were correctly classified.

##### CD and S with white background images

The PlantDoc dataset was utilized for training the proposed model by taking gls, and nlb images and evaluated on the CD & S dataset’s white background images of gls, and nlb. 100% of accuracy was achieved for nlb images while only 6.25% accuracy for gls images.

All the results of cross-dataset training for corn disease classification are presented in Table 23. Cross-dataset training for potato disease classification using the PlantVillage and PlantDoc datasets are presented in Table 24. Table 23 revealed that the proposed model achieved the best average classification accuracy which was 82.93% for corn disease classification when the model was trained on no-background images of CD & S dataset and evaluated on the images of the rest of the dataset. Table 24 revealed that the proposed model demonstrated modest accuracy for cross-dataset training of potato disease classification A comparison between existing studies and the proposed model’s performance is presented in Table 25. Table 25 revealed that our proposed model performed better as compared to the existing studies regarding cross-dataset training for corn disease classification. For potato disease classification, no such studies were reported previously using the PlantVillage and PlantDoc datasets. We presented a model here for potato disease classification which demonstrated modest accuracy using the abovementioned dataset for cross-dataset training.

**Table 23:** Results of cross-dataset training for corn disease classification in percentage using the proposed model.

Training dataset	PlantVillage	PlantDoc	Digipathos	NLB	CD & S	CD & S white background	CD & S Black background	Avg (%)
CD & S No background	gls(81.09) nlb(46.70) rust(-)	gls(89.71) nlb(25.52) rust(-)	nlb(99.36)	Nlb (89.22)	gls(82.03) nlb(77.87) nls(62.43)	gls (97.72) nlb (96.69) nls (99.64)	gls(99.62) nlb(97.52) nls(98.91)	82.93
PlantVillage	gls(-) nlb(-) rust(-)	gls(6.03) nlb(79.41) rust(19.27)	nlb(40.25)	Nlb (17.25)	gls(15.25) nlb(99.60) nls(-)	gls(20.85) nlb(100) nls(-)	gls(24.25) nlb(96.69) nls(-)	47.16
PlantDoc	gls(8.58) nlb(88.44) rust(35.40)	gls(-) nlb(-) rust(-)	nlb(21.55)	Nlb (22.30)	gls(5.25) nlb(99.40) nls(-)	gls(5.46) nlb(97.93) nls(-)	gls(6.25) nlb(100) nls(-)	44.60
CD & S	gls(84.00) nlb(4.00) rust(-)	gls(56.00) nlb(66.00) rust(-)	nlb(58)	Nlb (94)	gls(-) nlb(-) nls(-)	gls(69.00) nlb(63.00) nls(98.00)	gls(97.00) nlb(72.00) nls(56.00)	68.08

**Table 24:** Results of cross-dataset training for potato disease classification in percentage using the proposed model.

Training dataset	PlantVillage	PlantDoc
PlantVillage	Healthy(-), Early blight(-), Late blight(-)	Early blight(94.87%), Late blight(10.48%)
PlantDoc	Healthy(-), Early blight(74.50), Late blight(39.10)	Early blight(-), Late blight(-)

**Table 25:** Comparison table of existing study and the proposed model for cross-dataset training.

Study	Train/Test	Accuracy
^6^	PlantVillage/Field Field/PlantVillage	33.27% 65.69%
^24^	PlantVillage/IPM PlantVillage/Bing	45.95% 33.97%
^25^	PlantVillage/Field PlantVillage/Field	39.38% 72.03%
^16^	PlantVillage/PlantDoc	39.87%
^12^	CD & S No Background/PlantVillage, PlantDoc, Digipathos, NLB, CD & S, CD & S with White Background, and CD & S with Black Blackground	81.60% (Avg)
^12^	PlantVillage/PlantDoc, Digipathos, NLB, CD & S, CD & S with White Background, and CD & S with Black Blackground	45.36% (Avg)
^12^	CD & S/PlantVillage, PlantDoc, Digipathos, NLB, CD & S with White Background, and CD & S with Black Blackground	66.83% (Avg)
Proposed model	CD & S No Background/PlantVillage, PlantDoc, Digipathos, NLB, CD & S, CD & S with White Background, and CD & S with Black Blackground	82.93% (Avg)
	PlantVillage/PlantDoc, Digipathos, NLB, CD & S, CD & S with White Background, and CD & S with Black Blackground	47.16% (Avg)
	PlantDoc/PlantVillage, Digipathos, NLB, CD & S, CD & S White Background, and CD & S Black Blackground	44.60% (Avg)
	CD & S/PlantVillage, PlantDoc, Digipathos, NLB, CD & S White Background, and CD & S Black Blackground	68.08% (Avg)
	PlantVillage (Potato)/PlantDoc (Potato)	55%
	PlantDoc (Potato)/PlantVillage (Potato)	57%

## Limitations of the study

The variability found in actual agricultural situations, such as variations in illumination, background conditions, and image quality, may not be fully captured by the publically available datasets used in this work. Additionally, the dataset’s size and distribution may have an impact on the model’s capacity to generalize to new data. The current study is restricted to image-based disease categorization and does not take into account external elements like temporal fluctuations or environmental conditions. In order to increase the robustness and practical application of the suggested model, future research will concentrate on assessing it using real-time field data and more varied datasets.

## Conclusion and future work

This study started with the introduction and significance of plant disease classification. We reviewed various existing works related to corn and potato disease classification for intra and cross-dataset training. Then this study proposed an attention-based CNN model which was used for intra and cross dataset training using the PlantVillage, PlantDoc, Digipathos, NLB, and CD & S datasets. The proposed model obtained highest 99.38% of accuracy for potato disease classification using the PlantVillage dataset for intra-dataset training. For cross-dataset training, the proposed model attained highest 82.93% of average classification accuracy when trained on the no-background images of CD & S dataset and evaluated on the images of rest of the datasets for corn disease classification. This study contributed regarding generalization of deep learning models for plant disease classification which can be used in real world environment condition for plant disease classification. Further various other plant leaf datasets can be explored where intra and cross dataset training can be achieved with a good performance.

## Data Availability

The datasets used during the current study are publicly available. Additional data used and/or analyzed during the study are available from the corresponding author upon reasonable request.
